# Supplementary material for: Quantum Nature of Ubiquitous Vibrational Features Revealed for Ethylene Glycol
Source: J Chem Theory Comput. 2025 May 7;21(10):5208–20. doi: 10.1021/acs.jctc.5c00173 (PMC12121498; doi:10.1021/acs.jctc.5c00173)
Supplement: Supplementary file 1 [file ct5c00173_si_001.pdf]

Supporting Information: The quantum nature of ubiquitous vibrational features revealed for ethylene glycol

Apurba Nandi,[*,†] Riccardo Conte,[*,‡] Priyanka Pandey,[¶] Paul L. Houston,[§] Chen Qu,[‖] Qi Yu,[⊥] and Joel M. Bowman[*,¶]

†*Department of Physics and Materials Science, University of Luxembourg, L-1511, Luxembourg City, Luxembourg*

‡*Dipartimento di Chimica, Università degli Studi di Milano, via Golgi 19, 20133 Milano, Italy*

¶*Department of Chemistry and Cherry L. Emerson Center for Scientific Computation, Emory University, Atlanta, Georgia 30322, USA*

§*Department of Chemistry and Chemical Biology, Cornell University, Ithaca, New York 14853, USA and Department of Chemistry and Biochemistry, Georgia Institute of Technology, Atlanta, Georgia 30332, USA*

‖*Independent Researcher, Toronto, Ontario M9B0E3, Canada*

⊥*Department of Chemistry, Fudan University, Shanghai, 200438, P. R. China*

E-mail: apurba.nandi@uni.lu; riccardo.conte1@unimi.it; jmbowma@emory.edu

Details of the MULTIMODE Calculations

Below are the essential input parameters for the MULTIMODE calculations. More details are at https://scholarblogs.emory.edu/bowman/softwares/multimode/

NATOM,NSTAT,CONV,ICOUPL,ICOUPC,ISCFCI,IWHICH,IDISC,NROTTR,JMAX,INORM

10 -1 1.D-3 4 2 250 1

0 -9 0 0 0

MAXBAS

10 10 10 10 10 10 10 10 10 10 10 10 10 10 10

10 10 10 10 10 10 10 10 10 10 10 10 10 10 10

10 10 10 10 10 10 10 10 10 10 10 10 10 10 10

8 8 8 8 8 8 8 8 8 8 8 8 8 8 8

NBF,MBF,NVF

12 18 6 12

12 18 6 12

12 18 6 12

12 18 6 12

12 18 6 12

12 18 6 12

12 18 6 12

12 18 6 12

12 18 6 12

12 18 6 12

12 18 6 12

12 18 6 12

12 18 6 12

12 18 6 12

12 18 6 12

Table S1: Normal mode frequencies (cm^{-1}) for five low-lying conformers of ethylene glycol from unweighted fitting PES.

**Mode**	**tG+g-**		**g+G+g-**		**g-G+g-**		**tTt**		**g+Tg-**	
	MP2/TZ[a]	PES	MP2/TZ[a]	PES	MP2/TZ[a]	PES	MP2/TZ[a]	PES	MP2/TZ[a]	PES
1	168	170	168	166	100	87i	116	133	141	145
2	247	214	292	304	159	166	217	183	250	190
3	329	333	327	328	321	327	230	207	268	215
4	420	404	452	464	428	426	291	296	296	287
5	523	531	536	532	528	532	481	483	475	470
6	887	890	878	874	881	879	839	843	803	789
7	904	904	897	901	885	890	1009	1046	1027	1043
8	1066	1072	1059	1063	1051	1059	1076	1075	1074	1075
9	1100	1099	1073	1074	1063	1073	1094	1091	1090	1083
10	1130	1136	1122	1122	1126	1123	1167	1169	1109	1107
11	1178	1211	1204	1217	1198	1204	1190	1237	1140	1157
12	1269	1278	1246	1253	1257	1283	1235	1243	1320	1337
13	1296	1315	1374	1362	1383	1401	1288	1308	1339	1343
14	1384	1392	1377	1382	1389	1402	1319	1329	1370	1367
15	1420	1430	1406	1408	1404	1415	1409	1429	1404	1382
16	1455	1467	1435	1440	1422	1432	1487	1513	1433	1432
17	1516	1520	1511	1516	1508	1513	1541	1546	1521	1511
18	1524	1526	1521	1522	1512	1527	1551	1551	1535	1537
19	3053	3056	3026	3027	3071	3069	3057	3065	3067	3068
20	3058	3064	3070	3074	3074	3078	3064	3066	3076	3076
21	3114	3118	3134	3137	3140	3141	3102	3105	3124	3127
22	3149	3149	3159	3161	3148	3151	3127	3130	3150	3150
23	3808	3835	3794	3817	3845	3856	3857	3872	3840	3859
24	3856	3872	3831	3849	3846	3882	3858	3879	3842	3870
**MAE**		**10**		**6**		**9**		**13**		**12**

[a] From Table S-3 in Ref. 1

VCI frequencies

Table S2: VCI Frequency (cm^{-1}) and leading coefficients for $\mathbf{g^+G^+g^-}$ conformer.

Mode	Har. Freq.	CI Freq.	CI Coeff.	Coupling Modes
10	1121	1115	0.9923	ν_{10}
11	1215	1177	-0.9829, -0.0936, -0.0695	ν_{11}, ν_{12}, ν_{14}
12	1250	1214	0.9823, -0.1022, -0.0752	ν_{12}, ν_{11}, ν_{16}
13	1360	1315	-0.9765, -0.1711, 0.0833	ν_{13}, ν_{14}, ν_{11}
14	1379	1335	0.9240, -0.2688, -0.1694	ν_{14}, ν_{15}, ν_{13}
15	1405	1372	0.9304, 0.2980, 0.1838	ν_{15}, ν_{14}, ν_{16}
16	1437	1405	0.9611, -0.2273, 0.1066	ν_{16}, ν_{15}, ν_{14}
17	1514	1470	0.9961	ν_{17}
18	1519	1472	0.9967	ν_{18}
19	3027	2894	0.6762, 0.4132, 0.3474	ν_{19}, $(\nu_{16}+\nu_{18})$, $2\nu_{18}$
		2943	0.8374, -0.3496, 0.3443	$2\nu_{18}$, ν_{19}, ν_{21}
20	3073	2910	0.6971, -0.5907, -0.2296	$2\nu_{17}$, ν_{20}, $(\nu_{17}+\nu_{18})$,
		2971	0.6708, 0.4928, -0.4029	ν_{20}, $2\nu_{17}$, $(\nu_{17}+\nu_{18})$
21	3136	2943	0.8374, -0.3496, 0.3443	$2\nu_{18}$, ν_{19}, ν_{21}
		3003	-0.8705, 0.3152, -0.1917	ν_{21}, $2\nu_{18}$, $(\nu_{17}+\nu_{18})$
22	3160	3017	0.9119, -0.1847, -0.1522	$\nu_{22}, 2\nu_{17}, \nu_{20}$
23	3815	3614	0.9714, 0.1338, 0.0950	$\nu_{23}, (\nu_{11}+2\nu_{12}), \nu_{24}$
24	3847	3655	0.9385, -0.2650, -0.0923	$\nu_{24}, (2\nu_{11}+\nu_{13}), \nu_{23}$

Table S3: VCI Frequency (cm^{-1}) for $\mathbf{g}^{-}\mathbf{G}^{+}\mathbf{g}^{-}$ conformer.

Mode	Har. Freq.	CI Freq.	CI Coeff.	Coupling Modes
10	1123	1117	-0.9956	ν_{10}
11	1200	1168	0.9917	ν_{11}
12	1280	1237	-0.9818	ν_{12}
13	1401	1371	0.9564, -0.2311, -0.1472	ν_{13}, ν_{14}, ν_{15}
14	1403	1351	-0.8562, -0.3324, 0.2763	ν_{14}, ν_{15}, ν_{16}
15	1414	1353	-0.8889, -0.3144, 0.2662	ν_{15}, ν_{16}, ν_{14}
16	1428	1394	0.9004, 0.3561, -0.2146	ν_{16}, ν_{14}, ν_{15}
17	1513	1467	0.9897	ν_{17}
18	1526	1477	-0.9887	ν_{18}
19	3069	2908	0.6926, -0.5415, 0.2234	$2\nu_{17}$, ν_{19}, $(\nu_{17} + \nu_{18})$
		2964	0.5759, -0.5147, 0.3646	ν_{19}, $2\nu_{18}$, $(\nu_{17} + \nu_{18})$
20	3077	2920	0.6236, -0.5043, -0.4766	ν_{20}, $(\nu_{17} + \nu_{18})$, $2\nu_{18}$
		2983	0.6015, 0.5661, 0.3465	ν_{20}, $(\nu_{17} + \nu_{18})$, $2\nu_{18}$
21	3142	3001	-0.8345, 0.2630, -0.2177	ν_{21}, ν_{22}, $2\nu_{18}$
		3009	0.8584, 0.2584, -0.1771	ν_{22}, ν_{21}, ν_{19}
22	3150	3001	-0.8345, 0.2630, -0.2177	ν_{21}, ν_{22}, $2\nu_{18}$
		3009	0.8584, 0.2584, -0.1771	ν_{22}, ν_{21}, ν_{19}
23	3856	3657	0.6533, -0.4486, -0.2863	$(\nu_{10} + \nu_{11} + \nu_{15}), \nu_{23}, (2\nu_{11} + \nu_{15})$
		3660	0.7299, 0.5507, -0.1638	$\nu_{23}, (\nu_{10} + \nu_{11} + \nu_{15}), \nu_{24}$
		3669	0.6145, -0.4001, 0.2924	$(2\nu_{11} + \nu_{15}), \nu_{23}, (2\nu_{11} + \nu_{16})$
24	3876	3681	-0.7377, -0.3362, 0.2682	$3\nu_{12}, \nu_{24}, (2\nu_{12} + \nu_{15})$
		3683	0.8559, -0.3766, -0.1531	$\nu_{24}, 3\nu_{12}, (2\nu_{11} + \nu_{14})$

Table S4: CI Frequency (cm^{-1}) for **tTt** conformer.

Mode	Har. Freq.	CI Freq.	CI Coeff.	Coupling Modes
10	1168	1152	0.9943	ν_{10}
11	1236	1183	0.9857	ν_{11}
12	1241	1218	0.9941	ν_{12}
13	1306	1258	0.9863	ν_{13}
14	1328	1296	-0.9944	ν_{14}
15	1427	1387	-0.9884	ν_{15}
16	1513	1473	-0.9782	ν_{16}
17	1545	1503	-0.9788	ν_{17}
18	1549	1506	-0.9905	ν_{18}
		2894	-0.7185, -0.5062, -0.2092	$\nu_{19}, 2\nu_{16}, 2\nu_{17}$
19	3065	2961	0.8046, -0.4110, -0.2573	$2\nu_{16}, \nu_{19}, 2\nu_{17}$
		3016	-0.6320, -0.6191, 0.3689	$2\nu_{17}, 2\nu_{18}, \nu_{19}$
		2842	0.7807, 0.5193, 0.2552	$(\nu_{15}+\nu_{16}), \nu_{20}, (\nu_{15}+\nu_{17})$
20	3066	2942	0.7045, -0.4065, -0.3483	$\nu_{20}, (\nu_{15}+\nu_{16}), (\nu_{15}+\nu_{17})$
		3034	0.9191, -0.2876, -0.1614	$(\nu_{17}+\nu_{18}), \nu_{20}, (\nu_{16}+\nu_{18})$
21	3104	2968	-0.8986, -0.2165, -0.1774	$\nu_{21}, (\nu_{14}+\nu_{17}), (\nu_{14}+\nu_{16})$
22	3130	2990	0.9218, -0.1838, -0.1759	$\nu_{22}, (\nu_{12}+\nu_{16}), (\nu_{10}+\nu_{18})$
23	3870	3687	-0.8991, 0.3343, 0.1510	$\nu_{23}, \nu_{24}, (\nu_{23}+\nu_{24})$
24	3878	3690	0.9157, 0.3283, -0.1073	$\nu_{24}, \nu_{23}, 2\nu_{23}$

Table S5: CI Frequency (cm^{-1}) for $\mathbf{g^{+}Tg^{-}}$ conformer.

**Mode**	**Har. Freq.**	**CI Freq.**	**CI Coeff.**	**Coupling Modes**
10	1107	1106	-0.9899	ν_{10}
11	1154	1125	0.9856	ν_{11}
12	1337	1296	-0.7891, 0.5335, -0.2666	$\nu_{12}, \nu_{14}, \nu_{13}$
13	1343	1312	-0.9336, -0.2421, 0.1883	$\nu_{13}, \nu_{15}, \nu_{12}$
14	1364	1317	0.8238, 0.5424, -0.1183	$\nu_{14}, \nu_{12}, \nu_{15}$
15	1381	1341	-0.9503, 0.2143, -0.1889	$\nu_{15}, \nu_{13}, \nu_{12}$
16	1429	1405	-0.9934	ν_{16}
17	1507	1470	-0.9943	ν_{17}
18	1535	1492	0.9946	ν_{18}
19	3067	2790	-0.9130, -0.3094, 0.0815	$2\nu_{16}, \nu_{19}, (\nu_{12} + \nu_{18})$
		2912	-0.6502, -0.6075, 0.2754	$2\nu_{17}, \nu_{19}, 2\nu_{16}$
		2954	0.7031, -0.5006, -0.3991	$2\nu_{17}, \nu_{19}, 2\nu_{18}$
		2993	-0.7501, -0.4068, 0.4004	$2\nu_{18}, \nu_{21}, \nu_{19}$
20	3075	2910	-0.9348, 0.2284, 0.1855	$(\nu_{16} + \nu_{18}), \nu_{20}, (\nu_{17} + \nu_{18})$
		2925	-0.7167, -0.5180, -0.3171	$\nu_{20}, (\nu_{17} + \nu_{18}), (\nu_{16} + \nu_{18})$
		2996	-0.6766, 0.5223, 0.4347	$(\nu_{17} + \nu_{18}), \nu_{22}, \nu_{20}$
21	3127	2980	-0.8179, 0.4412, -0.1271	$\nu_{21}, 2\nu_{18}, \nu_{19}$
		2993	-0.7501, -0.4068, 0.4004	$2\nu_{18}, \nu_{21}, \nu_{19}$
22	3149	2996	-0.6766, 0.5223, 0.4347	$(\nu_{17} + \nu_{18}), \nu_{22}, \nu_{20}$
		3007	-0.7698, -0.4640, 0.2974	$\nu_{22}, (\nu_{17} + \nu_{18}), \nu_{19}$
23	3856	3666	-0.9619	ν_{23}
24	3869	3675	-0.9734	ν_{24}

Table S6: VCI frequency (cm^{-1}) comparison between PIP PES and the one from ref. 1 for conformer $\mathbf{tG^{+}g^{-}}$.

**Mode**	**Har. Freq.**	**VCI Freq.**	
		**PIP PES**	**Ramesh PES**[1]
10	1135	1119	1118
11	1207	1167	1155
12	1277	1246	1249
13	1314	1268	1271
14	1390	1340	1355
15	1429	1387	1392
16	1465	1429	1435
17	1519	1472	1477
18	1525	1478	1485
19	3056	2798<br>2918<br>2972<br>2981	2810<br>2917<br>2923<br>2986
20	3064	2836<br>2915<br>2961	2849<br>2912<br>2958
21	3118	2972<br>2981	2958<br>2967<br>2973
22	3148	3012	3013
23	3831	3629	3623
24	3871	3681	3674

Table S7: VSCF/VCI Energies (cm^{-1}) and VCI expansion coefficients for $\mathbf{tG^{+}g^{-}}$ conformer without Coriolis coupling.

Mode	Har. Freq.	CI Freq.	VCI Coeff.	Corresponding Modes
10	1135	1119	0.9957	ν_{10}
11	1207	1166	-0.9889, 0.0582, 0.0455	ν_{11}, ν_{12}, ν_{14}
12	1277	1245	0.9775, 0.1739, 0.0633	ν_{12}, ν_{13}, ν_{11}
13	1314	1268	0.9633, -0.1765, -0.1319	ν_{13}, ν_{12}, ν_{15}
14	1390	1339	-0.9345, -0.3040, -0.1419	ν_{14}, ν_{15}, ν_{13}
15	1429	1387	0.9355, -0.3206, 0.0953	ν_{15}, ν_{14}, ν_{13}
16	1465	1429	-0.9913, -0.0479, 0.0418	ν_{16}, ν_{18}, ν_{15}
17	1519	1471	0.9549, -0.2797, 0.0396	ν_{17}, ν_{18}, ν_{15}
18	1525	1477	0.9546, 0.2807, -0.0398	ν_{18}, ν_{17}, ν_{16}
		2797	-0.8310, -0.3904, -0.2719	$(\nu_{15} + \nu_{16})$, ν_{19}, $2\nu_{16}$
19	3056	2917	-0.5982, 0.4581, 0.3712	ν_{19}, $2\nu_{18}$, $2\nu_{16}$
		2971	0.6708, 0.3955, 0.3528	ν_{21}, $2\nu_{18}$, ν_{19}
		2980	0.5377, -0.5366, -0.4214	ν_{21}, $(\nu_{17} + \nu_{18})$, ν_{19}
20	3064	2836	0.8094, -0.3438, -0.2594	$2\nu_{16}$, $(\nu_{15} + \nu_{16})$, ν_{20}
		2905	-0.8229, 0.2845, -0.2218	$(\nu_{16} + \nu_{17})$, ν_{20}, $(\nu_{17} + \nu_{18})$
		2914	-0.5085, 0.5010, -0.4777	$(\nu_{16} + \nu_{17})$, $2\nu_{17}$, ν_{20}
		2960	0.5301, 0.4905, -0.3889	ν_{20}, $(\nu_{17} + \nu_{18})$, $2\nu_{18}$
21	3118	2971	0.6708, 0.3955, 0.3528	ν_{21}, $2\nu_{18}$, ν_{19}
		2980	0.5377, -0.5366, -0.4214	ν_{21}, $(\nu_{17} + \nu_{18})$, ν_{19}
22	3148	3011	0.8886, -0.1972, -0.1733	$\nu_{22}, 2\nu_{17}, (\nu_{17} + \nu_{18})$
23	3831	3629	-0.8159, -0.4973, 0.1774	$\nu_{23}, (2\nu_{10} + \nu_{15}), (2\nu_{10} + \nu_{14})$
24	3871	3681	-0.9622, -0.1575, 0.0869	$\nu_{24}, (\nu_{11} + 2\nu_{13}), \nu_{23}$

MULTIMODE Spectra

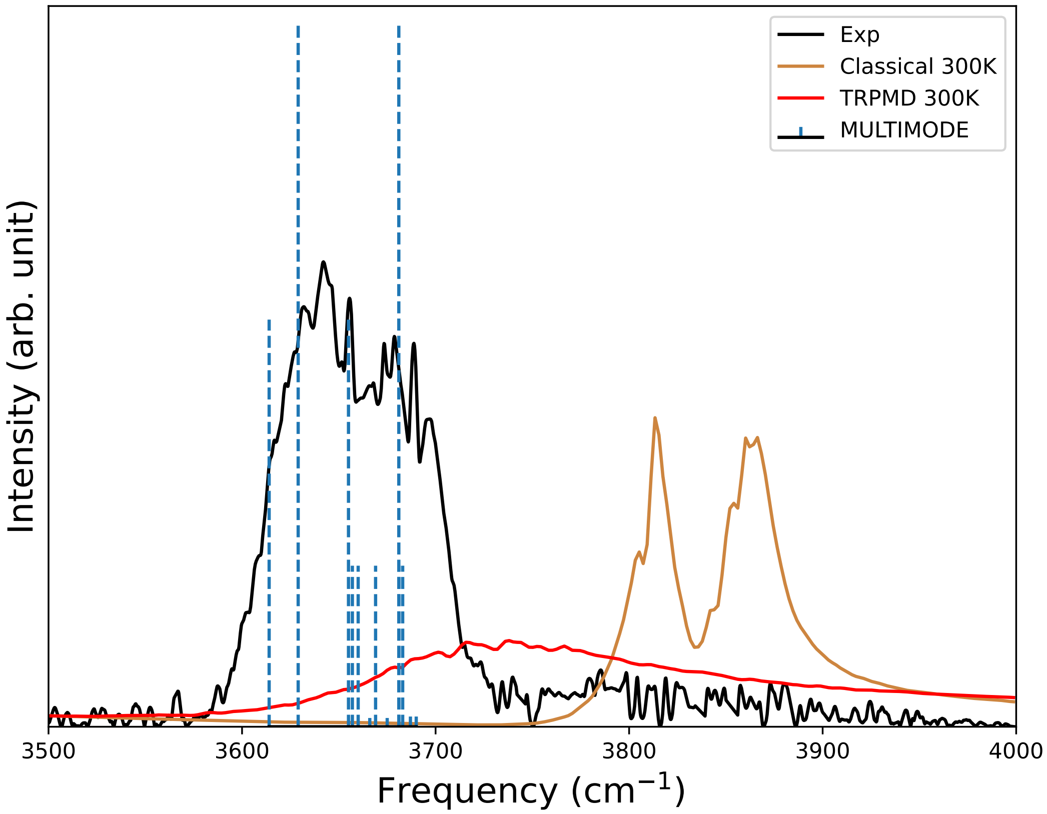

Figure S1: MULTIMODE eigenstates (blue dotted sticks) computed from VSCF/VCI calculations, power spectra at 300 K from Classical MD (orange curve) and TRPMD (red curve) simulations from Arandhara *et al*[1]compared with experimental IR spectra (black curve) from Das *et al*[2] for O−H stretching region.

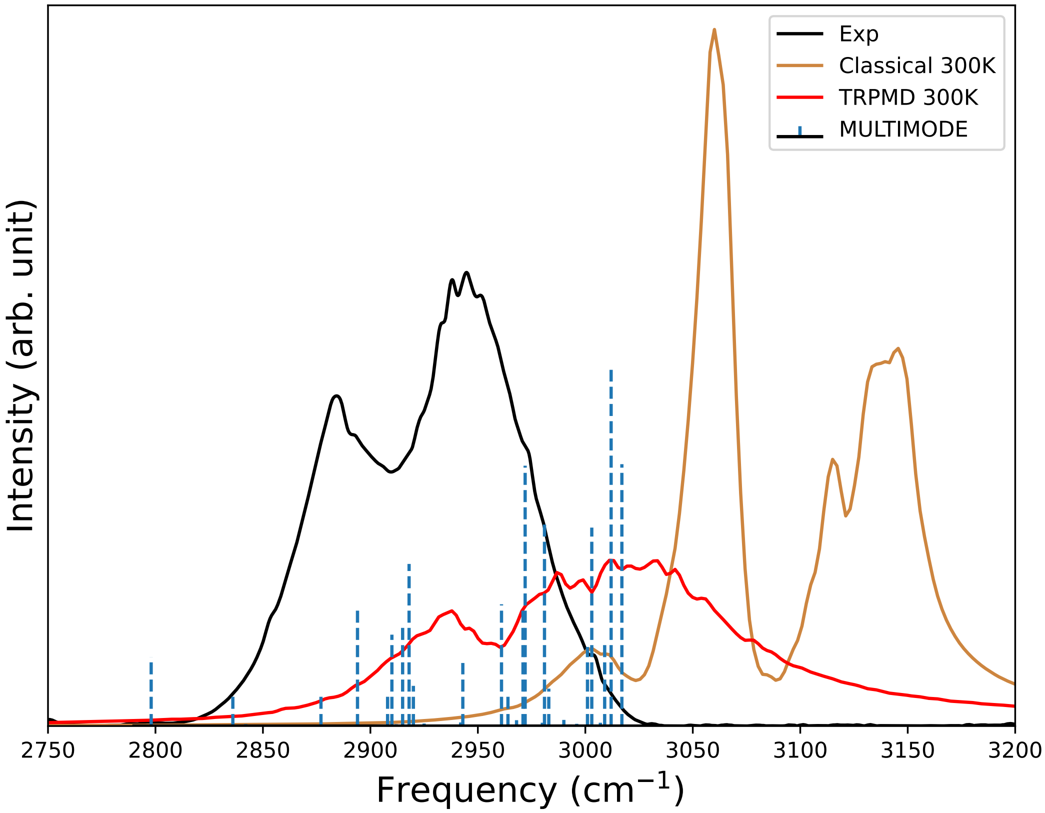

Figure S2: MULTIMODE eigenstates (blue dotted sticks) computed from VSCF/VCI calculations, power spectra at 300 K from Classical MD (orange curve) and TRPMD (red curve) simulations from Arandhara *et al*[1]compared with experimental IR spectra (black curve) from Das *et al*[2] for C−H stretching region.

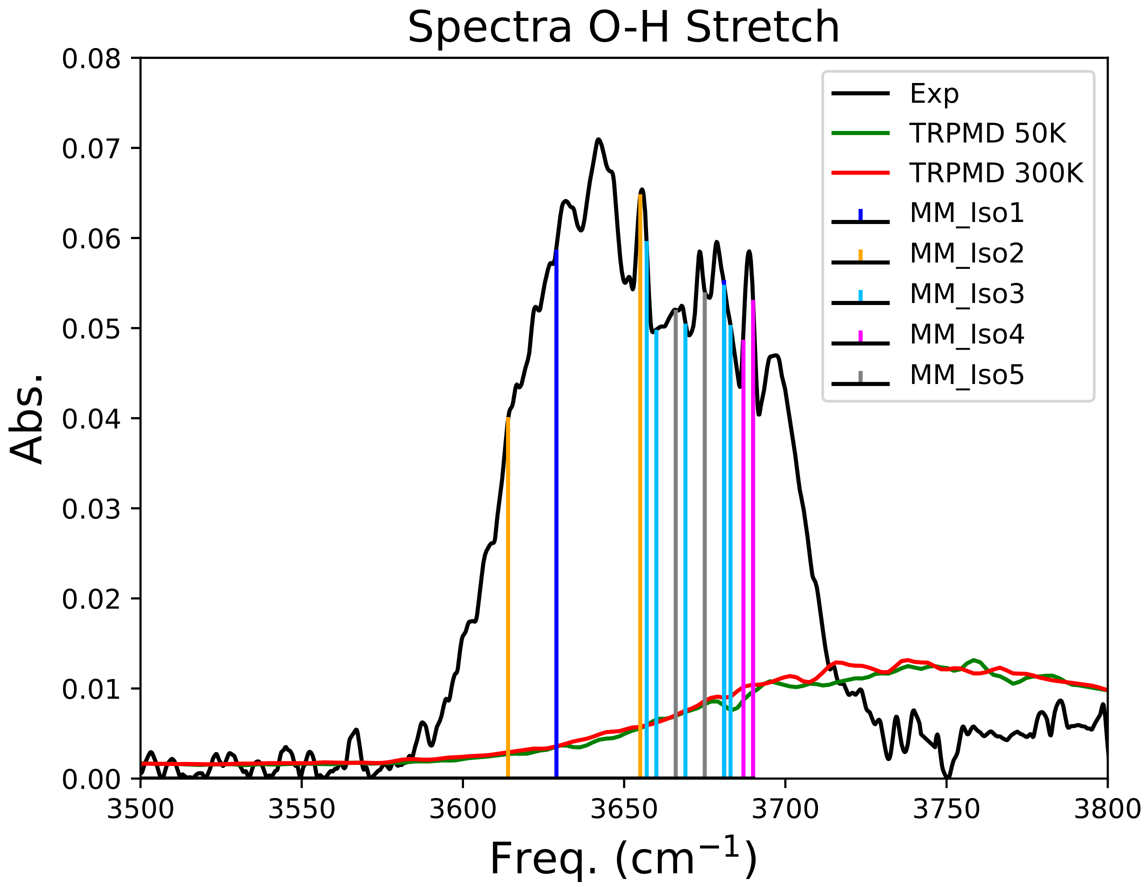

Figure S3: Contribution of each conformer to the VSCF/VCI spectrum, and TRPMD (at 50K and 300 K) simulations from Arandhara *et al*[1]compared with experimental IR spectra (black curve) from Das *et al*[2] for O−H stretching region.

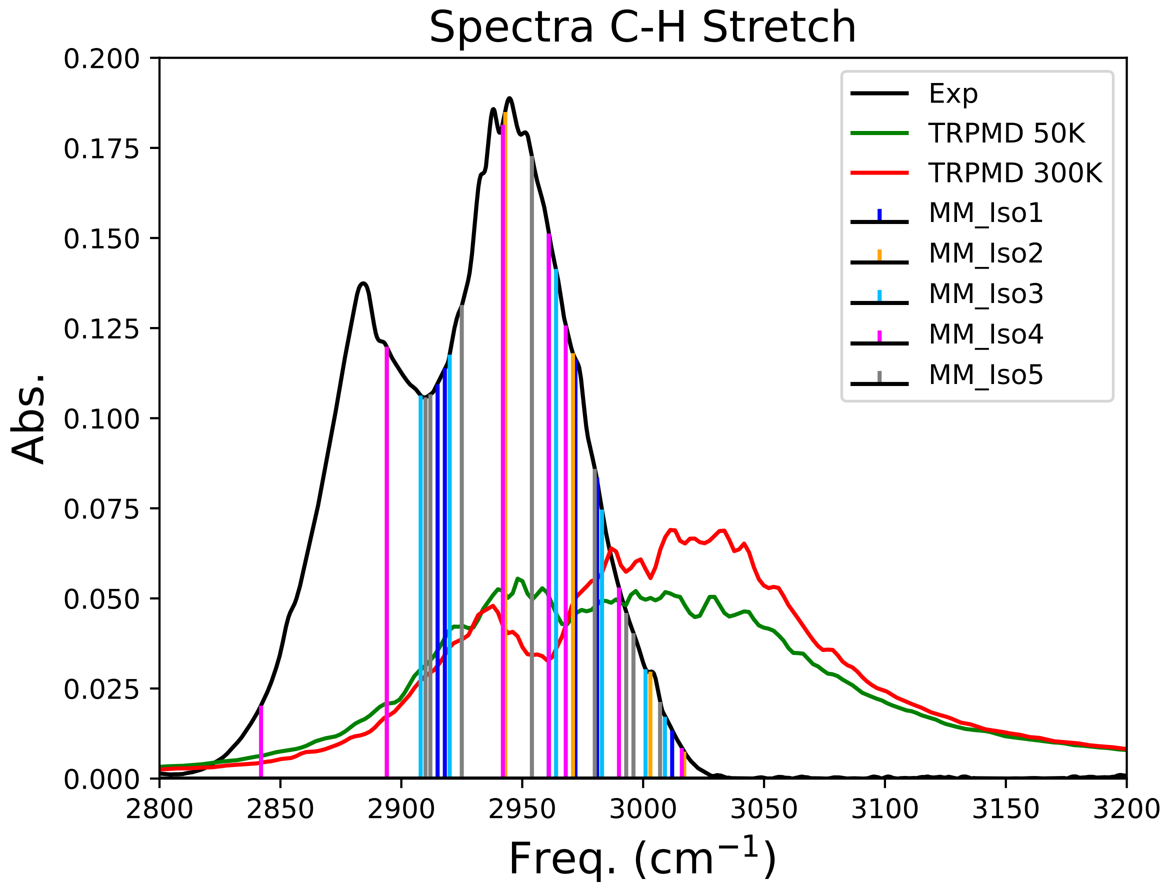

Figure S4: Contribution of each conformer to the VSCF/VCI spectrum, and TRPMD (at 50K and 300 K) simulations from Arandhara *et al*[1]compared with experimental IR spectra (black curve) from Das *et al*[2] for C−H stretching region.

AS-SCIVR CH-stretch band Fermi resonances

The following Figure shows that AS-SCIVR calculations performed on the global minimum **tG$^+$g$^-$** conformer are able to identify Fermi resonances at 2755 cm^{-1}and 2884 cm^{-1}, as well as a combination band of mode 19 with a low-frequency mode (arguably mode 3) found at 3176 cm^{-1}.

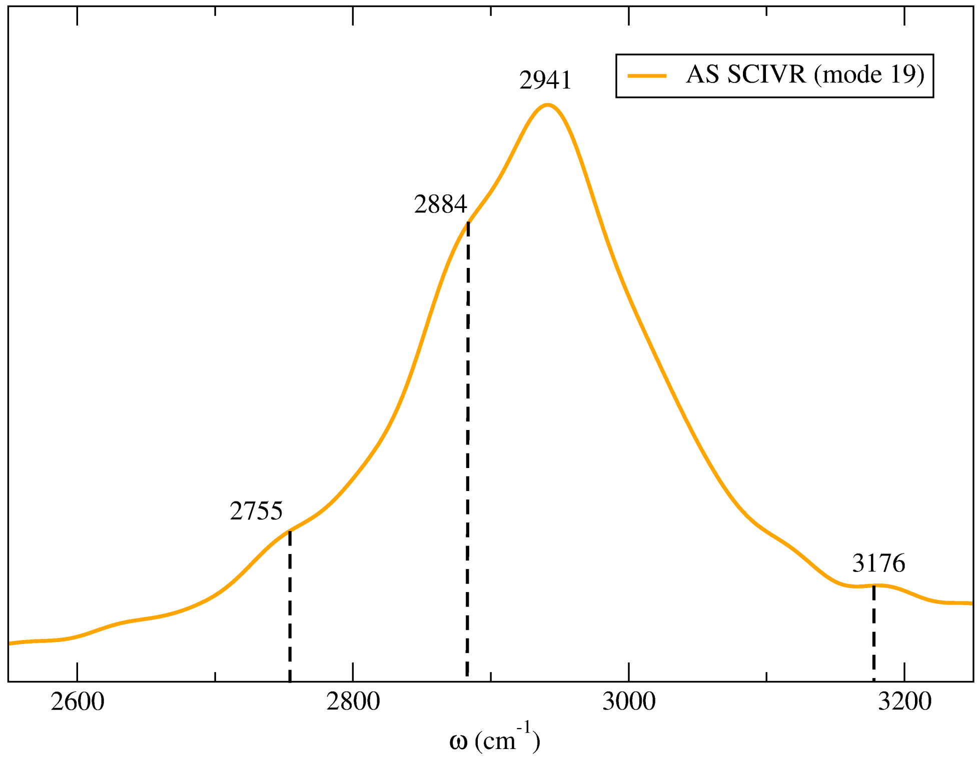

Figure S5: Fundamental frequency, Fermi resonances, and combination band involving mode 19 of the global minimum **tG$^+$g$^-$** conformer.

AS-SCIVR CH-stretch band: g^+Tg^- Conformer

The following Figure for the CH-stretch band shows a comparison between different power spectrum calculations and the experiment. AS-SCIVR calculations have been performed on modes 19-22 of the g^+Tg^- conformer.

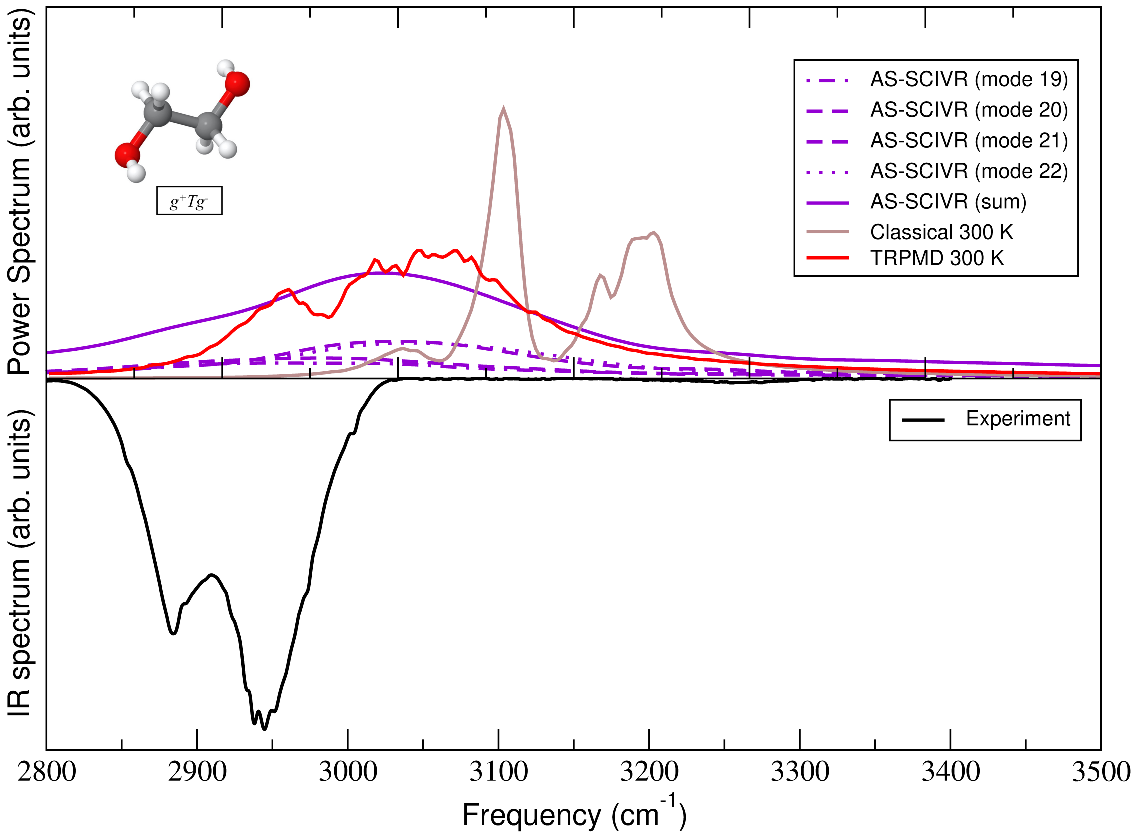

Figure S6: CH-stretch band. On top panel: AS-SCIVR results for modes 19-22 (violet, dashed and points) of the $\mathbf{g^+Tg^-}$ conformer, and their sum (violet, solid); TRPMD calculations at 300K (red); classical (brown). On bottom panel: experimental results (black).

AS-SCIVR CH-stretch band: tTt conformer

The following Figure for the CH-stretch band shows a comparison between different power spectrum calculations and the experiment. AS-SCIVR calculations have been performed on modes 19-22 of the tTt conformer.

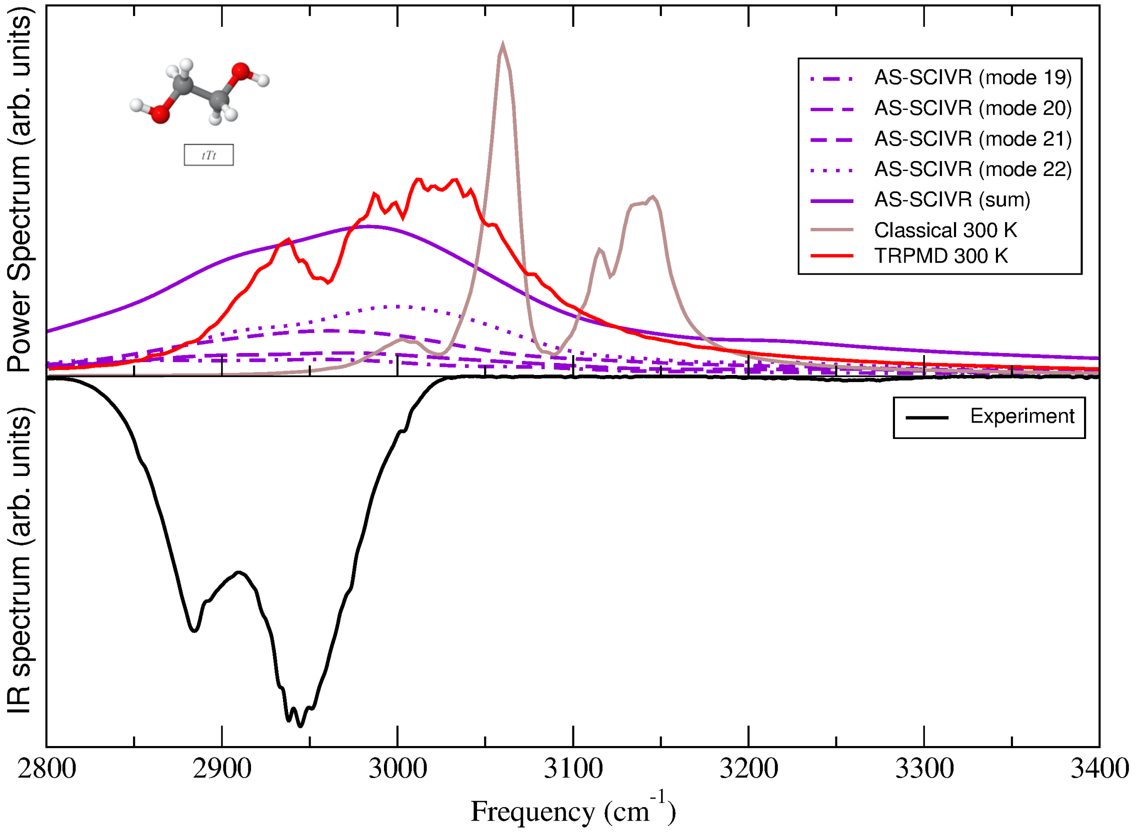

Figure S7: CH-stretch band. On top panel: AS-SCIVR results for modes 19-22 (violet, dashed and points) of the **tTt** conformer, and their sum (violet, solid); TRPMD calculations at 300K (red); classical (brown). On bottom panel: experimental results (black).

References

(1) Arandhara, M.; Ramesh, S. G. Nuclear quantum effects in gas-phase ethylene glycol. *Phys. Chem. Chem. Phys.* **2024**, *26*, 19529–19542.

(2) Das, P.; Das, P. K.; Arunan, E. Conformational Stability and Intramolecular Hydrogen Bonding in 1,2-Ethanediol and 1,4-Butanediol. *J. Phys. Chem. A* **2015**, *119*, 3710–3720.
